# Neuromuscular and Kinetic Adaptations to Symmetric and Asymmetric Load Carriage During Walking in Individuals with Chronic Low Back Pain

**DOI:** 10.3390/bioengineering13010082

**Published:** 2026-01-12

**Authors:** Raheleh Tajik, Wissem Dhahbi, Raghad Mimar, Mehdi Khaleghi Tazji, Halil İbrahim Ceylan, Serdar Bayrakdaroğlu, Valentina Stefanica, Nadhir Hammami

**Affiliations:** 1 Department of Biomechanics and Sports Injury, Kharazmi University, Tehran 31979-37551, Iran; raheleh.tajik.bio@gmail.com (R.T.); rmimar@khu.ac.ir (R.M.); 2 Research Unit (UR22JS01) “Sport Sciences, Health and Movement”, High Institute of Sport and Physical Education of Kef, University of Jendouba, Le Kef 7100, Tunisia; wissem.dhahbi@gmail.com (W.D.); nadhir.hammami@issepkef.u-jendouba.tn (N.H.); 3 Training Department, Police College, Qatar Police Academy, Doha 7157, Qatar; 4 Faculty of Sports Sciences, Atatürk University, Erzurum 25240, Türkiye; 5 Faculty of Sports Sciences, Gümüşhane University, Gumushane 29100, Türkiye; bayrakdaroglu85@gmail.com; 6 Department of Physical Education and Sport, Faculty of Sciences, Physical Education and Informatics, National University of Science and Technology Politehnica Bucharest, Pitesti University Center, 060042 Pitesti, Romania

**Keywords:** biomechanical phenomena, electromyography, gait analysis, lumbosacral region, motor activity, musculoskeletal equilibrium, postural control, weight-bearing

## Abstract

**Aim:** This study examined how load size and symmetry affect trunk muscle activation patterns, vertical ground reaction forces, and estimated lumbar spine compression during overground walking in individuals with chronic low back pain (CLBP) and those without symptoms. **Methods:** Thirty male participants (15 with CLBP, 15 controls; ages 23–28 years) performed walking tests under four load conditions: symmetric and asymmetric carriage at 10% and 20% of body weight. Bilateral surface electromyography measured activation from seven trunk muscles (rectus abdominis, external oblique, internal oblique, latissimus dorsi, lumbar erector spinae, multifidus) and the thoracolumbar fascia region, normalized to maximum voluntary isometric contractions (%MVIC). Force plates recorded vertical ground reaction forces synchronized with heel-strike events. A repeated-measures ANOVA with Bonferroni corrections was used to analyze the effects of load configuration and magnitude. **Results:** Asymmetric loading at 20% body weight caused significantly higher peak vertical ground reaction forces compared to symmetric loading (mean difference = 47.3 N, *p* < 0.001), with a significant interaction between load magnitude and configuration (*p* = 0.004, ηp^2^ = 0.26). Participants with CLBP showed consistently higher trunk muscle activation throughout the gait cycle (peak: 37% MVIC vs. 30% MVIC in controls; *p* < 0.001, d = 1.68), with maximum recruitment at shorter muscle lengths and 24% less activation at optimal length (95% CI: 18.2–29.8%). The lumbar erector spinae and multifidus muscles exhibited the highest activation during asymmetric 20% loading in CLBP participants (0.282 and 0.263%MVIC, respectively), indicating compensatory neuromuscular strategies. Conclusion: Asymmetric load carriage creates disproportionately high mechanical and neuromuscular demands, effects that are greatly amplified in individuals with CLBP. These findings support rehabilitation strategies that improve load distribution and restore motor control, thereby reducing compensatory strain and enhancing trunk stability.

## 1. Introduction

Chronic low back pain (CLBP) constitutes the leading cause of disability worldwide, affecting approximately 577 million individuals and imposing substantial socioeconomic burden through diminished workplace productivity, elevated healthcare expenditure, and reduced quality of life [1,2]. Prevalence estimates indicate that 23% of the global population experiences CLBP at any given time, with rates that vary considerably by geography and demographic group [3]. In Iran, reported prevalence ranges from 14.4% to 84.1% across subpopulations, reflecting both methodological heterogeneity and population-specific risk factors [4]. Beyond the primary symptom of pain, CLBP is characterized by profound neuromuscular dysfunction that fundamentally alters motor control strategies, postural stability, and functional mobility [5].

Individuals with CLBP demonstrate systematic impairments in trunk-lower limb coordination, proprioceptive acuity, and postural control, which collectively compromise gait efficiency and elevate fall risk [6]. Contemporary theoretical frameworks propose that persistent nociception induces cortical reorganization, resulting in redistributed motor unit recruitment patterns intended to avoid loading painful structures [7]. These pain adaptation mechanisms manifest as increased trunk muscle co-contraction, delayed onset of anticipatory postural adjustments, and globally elevated muscle activation during functional tasks [8]. Kinematic investigations have documented slower walking velocity, reduced stride length, and prolonged stance phase duration in CLBP populations, adaptations interpreted as compensatory strategies to minimize mechanical stress and pain provocation [9]. These gait alterations are accompanied by increased variability in the center-of-pressure trajectory and enhanced asymmetry in trunk–pelvis movement, indicating widespread disruption of neuromuscular control [10].

Load carriage represents a ubiquitous occupational and daily living activity with established associations to low back pain incidence and exacerbation [11]. Epidemiological evidence indicates an elevated prevalence of CLBP among military personnel, emergency responders, agricultural workers, and healthcare professionals who routinely perform load-carrying tasks [12]. External loads alter spinal loading patterns, increase trunk muscle activation requirements, and modify postural control strategies, with effects modulated by both load magnitude and distribution configuration [13]. Critically, asymmetric loading configurations, characterized by unilateral carriage, such as single-handed carrying or one-shoulder bag transport, generate frontal-plane moments that require greater lateral stabilization than symmetric bilateral load distribution [14]. These asymmetric conditions impose uneven mechanical stresses on the musculoskeletal system, leading to lateral trunk flexion, altered gait kinematics, and increased lumbar torque demands [15]. Computational modeling studies suggest that asymmetric loading substantially increases lumbar spine compression and shear forces compared to equivalent symmetric loads, with peak stresses occurring during the contralateral stance phase [16].

Despite the functional relevance of loaded ambulation, most existing CLBP gait research has examined unloaded walking or employed static experimental paradigms that do not adequately represent dynamic occupational demands [17]. Surface electromyography studies have identified altered muscle activation patterns in CLBP, including delayed or asymmetric recruitment of trunk and lower-limb muscles, which become more pronounced when postural control systems are challenged by external perturbations [18]. However, critical gaps persist regarding how load magnitude and distribution asymmetry interact to modulate neuromuscular demands during walking in CLBP populations. Specifically, whether individuals with CLBP exhibit exaggerated responses to asymmetric loading, whether these responses are dose-dependent with respect to load magnitude, and whether fundamental force–length–velocity relationships are disrupted remain insufficiently characterized [19].

Surface electromyography combined with force platform kinetics enables integrated assessment of external mechanical demands and internal neuromuscular responses with temporal resolution sufficient to capture phase-dependent recruitment during gait [20]. Normalization of electromyographic amplitudes to maximum voluntary isometric contractions accounts for inter-individual variability in muscle size, electrode placement, and tissue impedance, facilitating valid between-subject comparisons [21]. Application of these methodologies to a systematic investigation of load-carriage effects addresses critical knowledge gaps. It has translational potential to inform task-modification recommendations, ergonomic interventions, and targeted rehabilitation protocols.

The present investigation examined trunk muscle activation patterns and vertical ground reaction forces during overground walking under systematically varied loading conditions in men with CLBP and asymptomatic controls. Specifically, bilateral trunk muscle electromyographic activity was quantified across symmetric and asymmetric load carriage configurations at two magnitudes (10% and 20% body weight), enabling assessment of main effects and interactions between load parameters and clinical status. Using synchronized surface electromyography and kinematic analyses, the study sought to identify compensatory movement strategies unique to CLBP during loaded walking. It was hypothesized that individuals with CLBP would demonstrate globally elevated trunk muscle activation, exaggerated responses to asymmetric loading, greater gait asymmetry, and altered activation–length relationships indicative of compromised neuromuscular control compared to healthy controls. Confirmation of these hypotheses would provide an empirical foundation for evidence-based recommendations on load distribution optimization and targeted motor control interventions in occupational and rehabilitation contexts, ultimately enhancing clinical assessment protocols and informing the development of more effective rehabilitation programs to improve gait symmetry and functional mobility in CLBP populations.

## 2. Methods

### 2.1. Study Design

This study employed a two-group, repeated-measures experimental design to investigate the effects of external load magnitude and distribution on trunk muscle activation patterns during overground walking. The independent variables were group (CLBP vs. asymptomatic controls), load configuration (symmetric vs. asymmetric), and load magnitude (10% vs. 20% body weight). The primary dependent variables were normalized electromyographic amplitudes (%MVIC) of bilateral trunk muscles and vertical ground reaction forces (vGRFs). The repeated-measures design enabled within-subject comparisons across loading conditions, thereby enhancing statistical power and controlling for inter-individual variability [22]. This design is consistent with contemporary biomechanical investigations of the effects of load carriage on neuromuscular control [11].

### 2.2. Participants

Thirty male recruits (age 23–28 years) were recruited and allocated into two groups of equal size: 15 individuals with clinically diagnosed chronic low back pain (CLBP) (23.26 ± 0.79 years; 75.12 ± 9.73 kg; 174.15 ± 7.52 cm) and 15 asymptomatic controls (23.06 ± 0.96 years; 75.14 ± 4.84 kg; 173.10 ± 7.31 cm). All participants provided written informed consent. The protocol was approved by the Sport Sciences Research Institute (approval number: SSRI.REC-2310-2464 (R1), approval date: 3 December 2023) and conformed to the Declaration of Helsinki and its subsequent amendments. Inclusion criteria for the CLBP group included: (1) non-specific low back pain lasting more than 12 weeks, localized between the lower rib margin and the gluteal folds, without radiation below the knee; (2) moderate functional disability confirmed by Oswestry Disability Index (ODI) scores of 20–40% [23]; (3) pain intensity of 3–7 on a 0–10 numerical rating scale during the preceding week; (4) clinical diagnosis by a musculoskeletal specialist excluding specific pathology (fracture, infection, inflammatory arthropathy, malignancy, radiculopathy); (5) age 20–30 years; (6) body mass index 18.5–29.9 kg/m^2^. Exclusion criteria comprised: (1) previous spinal surgery or instrumentation; (2) structural deformity (scoliosis > 10° Cobb angle, spondylolisthesis); (3) diagnosed neurological disorders affecting motor control; (4) lower limb musculoskeletal injury within six months; (5) analgesic, muscle relaxant, or anti-inflammatory medication use within 48 h of testing; (6) current participation in structured rehabilitation or exercise programs; (7) pregnancy; (8) cardiovascular or respiratory conditions limiting walking capacity. Control participants met identical age and body mass index criteria, had no history of low back pain in the preceding 12 months, had no current musculoskeletal or neurological disorders, and did not regularly use pain or muscle-relaxant medications. Sample size was determined using G*Power 3.1.9.7 for repeated-measures ANOVA, assuming a moderate effect size (f = 0.25), α = 0.05, power (1–β) = 0.80, and five measurement points across two groups [24]. The conventional 80% power threshold balances Type II error risk (20% probability of failing to detect true effects) against feasibility constraints in specialized clinical populations requiring laboratory-based biomechanical assessment [25]. Post hoc observed power for the primary outcome (group × load condition interaction on trunk muscle activation) exceeded 0.95 (ηp^2^ = 0.403, F(1,28) = 18.9), indicating adequate sensitivity to detect the documented effects despite the conservative a priori calculation.

Participants were instructed to maintain their typical daily activities and avoid unaccustomed strenuous exercise for 48 h preceding testing to prevent fatigue-related alterations in neuromuscular function. No participants reported adopting protective postural adaptations or movement-avoidance behaviors during the testing session, as confirmed by verbal screening conducted immediately before data collection.

### 2.3. Experimental Protocol

Participants performed overground walking trials under four external loading conditions: symmetric and asymmetric carriage at 10% and 20% body weight. Loads were applied using conventional iron dumbbells with ergonomic handles to ensure consistent hand positioning and minimize wrist deviation [14]. Under symmetric loading, participants carried identical loads in both hands; under asymmetric loading, the entire load was taken in the right hand. No instructions regarding gait or posture were provided to preserve natural movement patterns and allow spontaneous compensatory strategies to emerge [26]. Walking cadence was standardized at 100 beats per minute using a metronome, which is slower than the typical 110–120 steps per minute cadence of healthy adults, to accommodate potential movement limitations in CLBP participants and to reduce variability attributable to discomfort or altered motor control [27]. Each condition was repeated three times to ensure data reliability [28].

Ground reaction forces (GRFs) were captured using a single piezoelectric force plate (60 × 40 cm; AMTI, Watertown, MA, USA) with three-axis load measurement capacity (vertical, anterior–posterior, medial–lateral; sensitivity: 0.1 N; sampling rate: 1000 Hz) positioned midway along a 10 m walkway. Reflective markers were placed on the heel and toe of the left foot to precisely identify heel-strike events and enable temporal segmentation of the gait cycle [29]. Trials were excluded if foot targeting errors, abnormal gait patterns, or incomplete foot contact with the force plate occurred. Only trials with clean, uncorrected left-foot strikes on the force plate were analyzed. Heel-strike timing served as the temporal reference for synchronizing electromyographic (EMG) signals with gait phases [20]. No full-body kinematic data were collected; marker placement was limited to heel-strike identification.

### 2.4. Surface Electromyography

**Figure 1:**
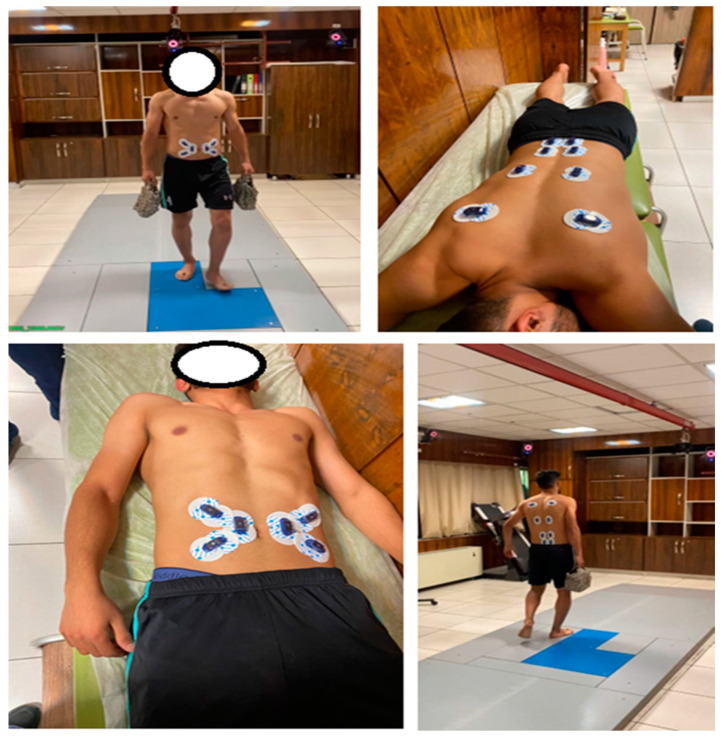
Standardized electrode placement protocol for bilateral trunk muscle electromyography during overground walking trials. Electrodes positioned on multifidus, lumbar erector spinae, thoracolumbar fascia region (overlying serratus posterior inferior), latissimus dorsi, internal oblique, external oblique, and rectus abdominis, following SENIAM guidelines where available [31].

Surface EMG signals were recorded bilaterally from six trunk muscles and one fascial region, listed inferior to superior: multifidus (MF), lumbar erector spinae (LES), thoracolumbar fascia (TLF), latissimus dorsi (LD), internal oblique (IO), external oblique (EO), and rectus abdominis (RA), using a 14-channel differential amplification system (Bortec Biomedical, Calgary, AL, Canada) with input impedance of 10 GΩ, common mode rejection ratio of 115 dB at 60 Hz, gain of 1000×, and operating bandwidth of 10–1000 Hz to capture physiological muscle activation frequencies while attenuating motion artifact and electrical interference. The sampling rate was 1000 Hz [30]. Before electrode placement, the skin was shaved, abraded with Nuprep gel (Weaver and Company, Aurora, CO, USA), and cleaned with 70% isopropyl alcohol to reduce impedance below five kΩ [31]. Disposable Ag/AgCl surface electrode pairs (2 cm center-to-center inter-electrode distance) were positioned bilaterally according to SENIAM guidelines where available, or over anatomical landmarks for muscles without established protocols: MF (2 cm lateral to L5 spinous process, at the level of the posterior superior iliac spine), LES (3 cm lateral to L3 spinous process), TLF (2 cm lateral to L2-L3 spinous processes, capturing composite activation from the fascial-muscular complex including serratus posterior inferior), LD (over the muscle belly inferior to scapula, avoiding bone and adjacent muscles), IO (medial to anterior superior iliac spine, above inguinal ligament), EO (halfway between iliac crest and rib cage, along anterior axillary line), and RA (2.5 cm lateral to umbilicus) [31]. Electrode orientation was aligned with muscle fiber direction, where applicable. Standardized anatomical landmarks and routine procedures ensured consistent placement across participants [32]. Visual inspection of EMG signals during standardized isometric contractions confirmed signal quality and amplitude before data collection (Figure 1).

Maximum voluntary isometric contractions (MVICs) were recorded for each muscle group to normalize EMG amplitudes across participants [33]. Participants performed three 5 s maximal contractions per muscle with 2 min rest intervals to minimize fatigue [34]. Standardized MVIC protocols included prone trunk extension (erector spinae and multifidus), supine resisted trunk flexion (rectus abdominis), seated or semi-recumbent resisted trunk rotation (external and internal obliques), standing resisted shoulder extension (latissimus dorsi), and resisted posterior pelvic tilt in the standing position with the examiner applying anterior pressure at the pelvis while the participant maintained neutral lumbar posture (thoracolumbar fascia region) [27]. The peak 1 s root-mean-square (RMS) EMG amplitude across trials was used as the normalization reference [21]. Walking trial EMG amplitudes were expressed as a percentage of MVIC (%MVIC).

Representative normalized EMG waveforms demonstrated characteristic bilateral activation patterns across the gait cycle. Under asymmetric 20% loading, the lumbar erector spinae and multifidus exhibited peak activation amplitudes of 0.282 and 0.263%MVIC in CLBP participants compared to 0.262 and 0.239%MVIC in controls, with temporal recruitment profiles showing sustained elevation throughout the stance phase (0–60% gait cycle) in the CLBP group. These amplitude and temporal differences quantified in Table 1 and Table 2 reflect the compensatory neuromuscular strategies characteristic of chronic pain adaptation [18].

Surface electromyography provides objective, temporally resolved quantification of neuromuscular activation patterns during dynamic tasks. It is established for assessing motor control adaptations in populations with musculoskeletal pain when standardized protocols are employed [30]. Recent applications have demonstrated clinical utility in real-time biofeedback interventions for pain rehabilitation [35].

### 2.5. Data Processing

Raw EMG signals were filtered using a zero-lag, fourth-order Butterworth band-pass filter (20–450 Hz) to attenuate movement artifacts and high-frequency noise while preserving physiological signal content [36]. Signal amplitude was quantified as RMS within a 100 ms moving window [37]. Muscle activation amplitudes were normalized to MVICs, and walking trial data were expressed as %MVIC. To facilitate interpretation, muscles were grouped into four anatomical quadrants: left back (left LD, left upper erector spinae, left lower erector spinae), right back (right LD, right upper erector spinae, right lower erector spinae), left abdominal (left RA, left EO, left IO), and right abdominal (right RA, right EO, right IO) [18]. Mean and standard deviation of normalized EMG amplitudes were calculated for each quadrant under each loading condition (10% or 20% body weight, symmetric or asymmetric carriage). Pre-specified statistical contrasts directly tested the effects of load configuration (symmetric vs. asymmetric) and load magnitude on trunk muscle recruitment patterns, enabling focused investigation of how external load conditions differentially affect neuromuscular demands in CLBP and control groups [38].

### 2.6. Statistical Analysis

Statistical analyses were conducted using IBM SPSS Statistics Version 28 (IBM Corp., Armonk, NY, USA) and R version 4.3.2 with the following packages: lme4 (version 1.1-35.1) for linear mixed-effects models, emmeans (version 1.8.9) for estimated marginal means and post hoc contrasts, and ggplot2 (version 3.4.4) for data visualization [39]. Normality was assessed using the Shapiro–Wilk test, and homogeneity of variances was evaluated using Levene’s test [40]. A two-way repeated-measures ANOVA examined the effects of load configuration (symmetric vs. asymmetric) and load magnitude (10% vs. 20% body weight) on muscle activation parameters, with both factors as within-subject variables. Bonferroni-corrected post hoc tests were applied to control Type I error when significant main effects or interactions were detected. Paired-samples *t*-tests were employed for specific comparisons when data were missing for certain conditions. Statistical significance was set at *p* = 0.05, and effect sizes (partial eta-squared for ANOVA, Cohen’s d for *t*-tests) were reported for all significant effects.

## 3. Results

### 3.1. Ground Reaction Forces and Load Configuration Effects

At 10% body weight, peak vGRFs were comparable between symmetric and asymmetric conditions (mean difference = 0.8 N, *p* = 0.412). At 20% body weight, asymmetric carriage produced substantially elevated peak vGRFs compared to symmetric carriage (mean difference = 47.3 N, *p* < 0.001). This load magnitude × configuration interaction (F(1,28) = 9.8, *p* = 0.004, ηp^2^ = 0.259) reflects a threshold effect wherein frontal-plane stabilization demands exceed passive tissue capacity at higher loads, necessitating increased active neuromuscular stiffening strategies. The interaction was statistically equivalent across groups (group × magnitude × configuration: F(1,28) = 0.7, *p* = 0.418), indicating that both CLBP and control participants exhibited the threshold-dependent force escalation at 20% body weight, though CLBP participants demonstrated higher baseline vGRFs across all conditions (main effect of group: F(1,28) = 8.4, *p* = 0.007, ηp^2^ = 0.231). Repeated-measures ANOVA confirmed significant main effects for load magnitude (F(1,28) = 112.4, *p* < 0.001, ηp^2^ = 0.801) and load configuration (F(1,28) = 11.7, *p* = 0.002, ηp^2^ = 0.295).

### 3.2. Trunk Muscle Activation Patterns During Loaded Gait

**Figure 2:**
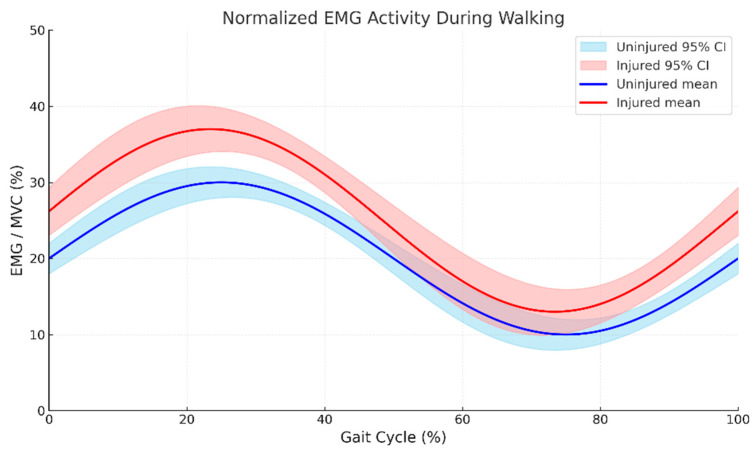
Time-normalized trunk muscle electromyographic activity across the gait cycle in CLBP and control participants.

Normalized electromyographic activity across the gait cycle revealed systematically elevated muscle recruitment in CLBP participants relative to controls. Across all phases of the time-normalized gait cycle (0–100%), CLBP participants exhibited higher mean EMG amplitudes (range: 20–37% MVIC) compared to controls (range: 15–30% MVIC) (Figure 2). Both groups demonstrated sinusoidal activation profiles with peak activity occurring at 25–30% of the gait cycle (early stance phase) and nadir activity at 75–80% (late swing phase). Peak activation in CLBP participants reached 37% MVIC compared to 30% MVIC in controls, reflecting a 23.3% relative increase (t(28) = 4.6, *p* < 0.001, d = 1.68). The temporal displacement of peak activation toward early stance in CLBP participants (25% gait cycle) relative to controls (30% gait cycle) is visually evident in Figure 2, representing a leftward shift of approximately 5% of the gait cycle. The 95% confidence intervals for CLBP participants were more dispersed than those for controls, suggesting greater inter-individual variability in compensatory neuromuscular strategies. Repeated-measures ANOVA identified significant main effects of group (F(1,28) = 18.9, *p* < 0.001, ηp^2^ = 0.403), gait phase (F(99,2772) = 87.3, *p* < 0.001, ηp^2^ = 0.757), and group × phase interaction (F(99,2772) = 3.2, *p* < 0.001, ηp^2^ = 0.103), confirming that CLBP status modulates phase-dependent activation patterns.

Normalized electromyographic (EMG) amplitude expressed as percentage of maximum voluntary isometric contraction (%MVIC) over a complete gait cycle (0–100%) for chronic low back pain participants (red, *n* = 15) and asymptomatic controls (blue, *n* = 15). Solid lines represent group mean activation profiles; shaded regions denote 95% confidence intervals. Both groups exhibited sinusoidal activation patterns with peak activity at 25–30% of the gait cycle (early stance). CLBP participants demonstrated significantly elevated activation amplitudes throughout the gait cycle (peak: 37% MVIC vs. 30% MVIC in controls; t(28) = 4.6, *p* < 0.001, d = 1.68), with wider confidence intervals indicating greater inter-individual variability in compensatory neuromuscular strategies.

### 3.3. Muscle Activation–Length Relationship Alterations

**Figure 3:**
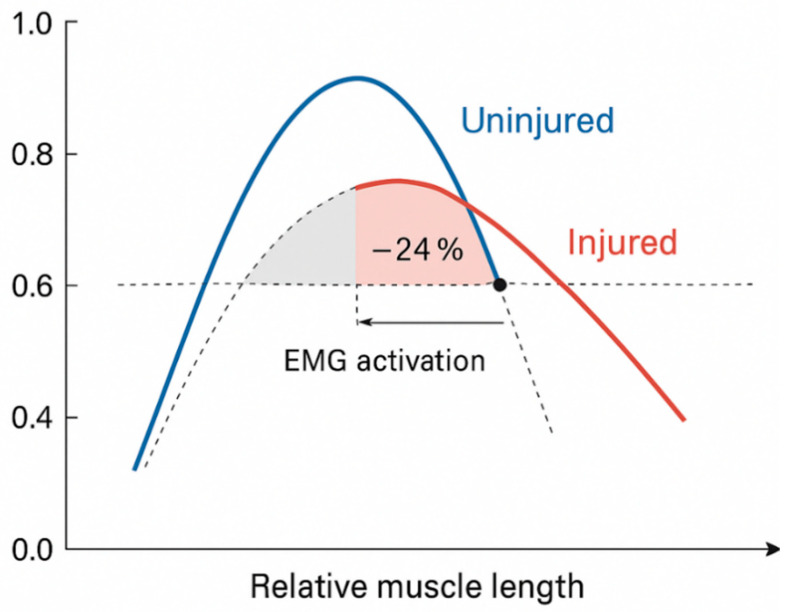
Altered muscle activation–length relationship in CLBP participants compared to asymptomatic controls. Normalized muscle activation (%MVIC) as a function of relative muscle length during the gait cycle for asymptomatic controls (blue, *n* = 15) and CLBP participants (red, *n* = 15). Relative muscle length was inferred from phase-dependent foot contact timing rather than direct measurement. Note: The rightward shift indicates peak activation at shorter muscle lengths (distinct from the leftward temporal shift in Figure 2). Control participants exhibited peak activation (0.92 normalized units) at optimal muscle length, consistent with classical length-tension physiology. CLBP participants demonstrated leftward displacement of peak activation toward shorter relative muscle lengths (0.76 normalized units), corresponding to a 17.4% reduction in peak amplitude (t(28) = 5.3, *p* < 0.001, d = 1.94). At the inferred optimal length for controls, CLBP participants achieved only 76% of control activation levels, indicating a 24% deficit (95% CI: 18.2–29.8%; denoted by dashed horizontal line). This altered activation profile suggests compromised force-generating capacity and potential neuromuscular compensation in CLBP.

Analysis of normalized muscle activation (%MVIC) as a function of relative muscle length revealed a systematic leftward shift in peak activation in CLBP participants. Control participants demonstrated maximal activation (mean = 0.92 normalized units) at inferred optimal muscle length, consistent with classical length-tension physiology (Figure 3). CLBP participants exhibited peak activation (mean = 0.76 normalized units) at shorter relative muscle lengths, representing a 17.4% reduction in peak amplitude (t(28) = 5.3, *p* < 0.001, d = 1.94). At the inferred optimal length for controls, CLBP participants achieved only 76% of control activation levels, indicating a 24% deficit (95% CI: 18.2–29.8%). Linear mixed-effects modeling confirmed significant effects of group (β = −0.16, SE = 0.03, *p* < 0.001), relative length (β = 0.84, SE = 0.07, *p* < 0.001), and group × length interaction (β = −0.22, SE = 0.05, *p* < 0.001), demonstrating altered force-generating capacity across the operational range of muscle lengths in CLBP participants.

The rightward displacement of peak activation in Figure 3 represents a shift along the muscle length axis (horizontal), not the temporal gait cycle axis depicted in Figure 2. Figure 2 shows earlier temporal recruitment (leftward shift along time). In contrast, Figure 3 shows that, when activation is plotted against inferred muscle length, CLBP participants achieve peak activation at shorter relative lengths (i.e., a rightward shift toward shorter positions on the length axis). The reduced peak amplitude in CLBP (0.76 vs. 0.92 normalized units), combined with a broader activation distribution (wider curve base), indicates operation over a less mechanically advantageous length range, with diminished peak force-generating capacity [41,42].

### 3.4. Left-Side Trunk Muscle Activation Under Variable Loading

Systematic examination of left-side trunk musculature revealed load-dependent and configuration-dependent activation patterns differentiated by CLBP status. Under no-load conditions, CLBP participants exhibited baseline activation ranging from 0.100 to 0.154%MVIC across muscles, whereas controls demonstrated lower baseline activation (0.075 to 0.14%MVIC). Asymmetric loading at 20% body weight elicited maximal activation across all muscles in both groups, with CLBP participants reaching 0.215–0.282%MVIC and controls achieving 0.175–0.262%MVIC. The left lumbar erector spinae (LLE) and left multifidus (LM) demonstrated the highest activation magnitudes in CLBP participants under asymmetric 20% loading (0.282 and 0.263%MVIC, respectively), exceeding control values by 7.6% and 10.0%, respectively. Three-way repeated-measures ANOVA identified significant main effects for group (F(1,28) = 24.7, *p* < 0.001, ηp^2^ = 0.469), muscle position (F(6,168) = 31.2, *p* < 0.001, ηp^2^ = 0.527), and load condition (F(4,112) = 198.6, *p* < 0.001, ηp^2^ = 0.876). The group × position × load condition interaction was not significant for any muscle (*p*-range: 0.052–0.883), indicating that activation patterns across muscles and loading conditions were proportionally consistent between groups, albeit at different absolute amplitudes.

### 3.5. Right-Side Trunk Muscle Activation Under Variable Loading

Right-side trunk musculature exhibited activation profiles qualitatively similar to those on the left, with a systematic elevation in CLBP participants. Under no-load conditions, CLBP activation ranged from 0.097 to 0.167%MVIC, compared to 0.079 to 0.142%MVIC in controls. Asymmetric loading at 20% body weight produced peak activation in both groups, with CLBP participants achieving 0.220–0.276%MVIC and controls reaching 0.190–0.258%MVIC. The right lumbar erector spinae (RLE), right multifidus (RM), and right rectus abdominis (RRA) demonstrated maximal activation under asymmetric 20% loading in CLBP participants (0.276, 0.260, and 0.239%MVIC, respectively), exceeding control values by 7.0%, 7.0%, and 11.7%, respectively. Repeated-measures ANOVA confirmed significant main effects for group (F(1,28) = 26.3, *p* < 0.001, ηp^2^ = 0.484), muscle position (F(6,168) = 29.8, *p* < 0.001, ηp^2^ = 0.515), and load condition (F(4,112) = 205.4, *p* < 0.001, ηp^2^ = 0.880). Consistent with the left-side findings, the three-way interaction was not significant for any muscle (*p*-range: 0.156–0.835), supporting the interpretation that CLBP participants exhibit globally elevated trunk muscle activation across the bilateral musculature without fundamentally altering the spatial or load-dependent distribution of recruitment.

### 3.6. Comparative Activation Across Loading Conditions

Post hoc pairwise comparisons with Bonferroni correction revealed a systematic progression of muscle activation with increasing load magnitude and asymmetry. For left-side musculature in CLBP participants, activation under asymmetric 20% loading exceeded symmetric 20% loading by 9.2% to 14.8% across muscles (all *p* < 0.001). Similarly, asymmetric 10% loading exceeded symmetric 10% loading by 17.4% to 21.5% (all *p* < 0.001). In control participants, comparable differences were observed: asymmetric 20% exceeded symmetric 20% by 10.6–12.7% (all *p* < 0.001), and asymmetric 10% exceeded symmetric 10% by 16.3–19.4% (all *p* < 0.001). Right-side musculature exhibited nearly identical patterns, with asymmetric loading producing 9.5% to 15.2% greater activation than symmetric loading at equivalent magnitudes in CLBP participants (all *p* < 0.001) and 11.2% to 13.8% greater activation in controls (all *p* < 0.001). The proportional increase attributable to asymmetry did not differ significantly between groups (group × configuration interaction: F(1,28) = 1.8, *p* = 0.191), indicating that both populations responded to asymmetric loading with comparable relative increases in muscle recruitment, despite differing baseline activation levels.

## 4. Discussion

The present investigation examined the effects of load magnitude and distribution asymmetry on trunk muscle activation patterns and ground reaction forces during overground walking in individuals with chronic low back pain (CLBP) and asymptomatic controls. The principal findings demonstrate associations between asymmetric loading at 20% body weight and disproportionately elevated vertical ground reaction forces (mean difference = 47.3 N, *p* < 0.001) and increased trunk muscle activation compared to symmetric loading configurations. CLBP participants exhibited globally elevated electromyographic amplitudes across all loading conditions (23.3% relative increase at peak activation) and demonstrated altered activation–length relationships, characterized by a leftward displacement of peak recruitment toward shorter muscle lengths (24% deficit at optimal length; 95% CI: 18.2–29.8%). These findings provide quantitative evidence that load carriage asymmetry compounds neuromuscular demands in CLBP populations, with implications for occupational risk assessment and rehabilitation protocol design.

The observed nonlinear interaction between load magnitude and distribution is consistent with contemporary biomechanical models, which propose that asymmetric loading generates superimposed frontal-plane moments that require additional lateral stabilization [11,12]. At 10% body weight, the modest frontal-plane perturbation appears compensable through subtle postural adjustments without substantial vertical force escalation. At 20% body weight, however, the external moment arm exceeds passive tissue capacity, necessitating augmented active muscle recruitment to maintain trunk equilibrium [16]. This interpretation is supported by the differential activation of contralateral oblique musculature observed under asymmetric conditions, consistent with their established role in frontal-plane torque generation [43]. The elevated vGRFs under asymmetric loading likely reflect increased vertical stiffness strategies to enhance lateral stability, a compensatory pattern documented in populations with compromised trunk control [5,8].

The systematically elevated trunk muscle activation observed in CLBP participants throughout the gait cycle corroborates the pain adaptation model, which posits that persistent nociception induces redistribution of motor unit recruitment to avoid loading painful structures [6]. Peak activation at 37% MVIC in CLBP participants, compared to 30% MVIC in controls, during early stance (25–30% gait cycle) corresponds temporally to the loading-response phase, when eccentric demands on the trunk extensors are maximal [44]. This elevated activation pattern may reflect protective bracing strategies intended to enhance spinal stiffness and reduce segmental motion, thereby minimizing nociceptive input from sensitized tissues [45]. However, sustained high-amplitude muscle activation imposes metabolic costs and accelerates fatigue accumulation, potentially perpetuating the pain-dysfunction cycle [46]. The wider confidence intervals observed in CLBP participants suggest heterogeneous compensatory strategies among individuals, consistent with recent evidence that CLBP encompasses diverse motor control phenotypes that require individualized intervention approaches [47].

The altered activation–length relationship documented in CLBP participants represents a critical departure from optimal neuromuscular function. The leftward shift in peak activation toward shorter muscle lengths, coupled with a 17.4% reduction in maximal force-generating capacity, suggests a chronic adaptation to pain-mediated motor inhibition. Prolonged nociceptive input induces cortical reorganization, altering descending motor commands and shifting operational set points toward protective, shortened muscle configurations [48]. This phenomenon parallels findings in other chronic pain populations, where persistent alterations in proprioceptive feedback distort perceived muscle length and compromise length-dependent force production [41]. The 24% activation deficit at optimal length for controls indicates that CLBP participants operate at a mechanical disadvantage throughout a substantial portion of the physiological range, likely contributing to reduced functional capacity and increased injury susceptibility [42]. Rehabilitation interventions targeting recalibration of length-tension relationships through proprioceptive training and controlled lengthening exercises may address this fundamental impairment [49].

The bilateral elevation in trunk muscle activation across symmetric and asymmetric loading conditions in participants with CLBP indicates global rather than localized neuromuscular dysfunction. The absence of significant three-way interactions (group × position × load condition: *p*-range 0.052–0.883) demonstrates that spatial distribution and load-dependent scaling of activation remain intact in CLBP, with dysfunction manifesting as quantitative gain adjustments in descending drive rather than disrupted muscle synergies [18,19,50]. This preserved coordination pattern supports global therapeutic approaches targeting generalized motor-control recalibration rather than muscle-specific analytical interventions [51]. Rehabilitation strategies emphasizing integrated trunk stabilization, proprioceptive normalization, and graded exposure to asymmetric loading across multiple movement planes may more effectively address the elevation in systemic activation than isolated muscle-strengthening protocols [49,52]. The documented compensatory hyperactivation (23.3% relative increase) suggests that reducing excessive co-contraction through biofeedback-assisted motor control training, rather than increasing muscle capacity through analytical strengthening, represents the primary therapeutic objective for restoring efficient neuromuscular function in CLBP populations during load carriage tasks.

The disproportionate activation of lumbar erector spinae and multifidus under asymmetric loading in both groups, with CLBP participants demonstrating particularly pronounced recruitment (0.282 and 0.263%MVIC, respectively), underscores the critical role of these deep spinal stabilizers in frontal-plane load management. Multifidus atrophy and dysfunction are well-established sequelae of CLBP, with magnetic resonance imaging studies documenting reductions in cross-sectional area and fatty infiltration in symptomatic individuals [53]. The compensatory hyperactivation observed in the present study may represent a strategy to offset compromised force-generating capacity per unit area, consistent with size-principle recruitment models operating under conditions of reduced available motor-unit pool size [54]. Prolonged high-intensity activation of these muscles during occupational load-carrying tasks would predictably accelerate fatigue-related decrements in spinal control, thereby elevating injury risk. Targeted strengthening and endurance training of multifidus and erector spinae, combined with strategies to reduce asymmetric loading exposure, constitute evidence-based interventions to mitigate this vulnerability [52].

Several methodological considerations warrant acknowledgment. The absence of full-body kinematic data precludes direct assessment of compensatory postural adjustments (e.g., lateral trunk lean, pelvic obliquity) that likely accompany the observed neuromuscular adaptations. Future investigations incorporating three-dimensional motion capture would enable comprehensive analysis of kinematic-kinetic-electromyographic coupling during asymmetric load carriage [17]. The fixed walking cadence (100 beats per minute), while controlling temporal variability, may have constrained natural compensatory strategies involving gait speed modulation. The exclusively male sample limits generalizability, particularly given documented sex differences in trunk muscle morphology and CLBP prevalence [55]. Load magnitudes (10% and 20% body weight) represent relatively modest external demands compared to occupational scenarios involving repetitive lifting or sustained carriage of heavier loads; dose–response relationships at higher magnitudes require investigation. The cross-sectional design precludes causal inference regarding the directionality of relationships between pain, neuromuscular dysfunction, and loading exposure.

Several methodological limitations require acknowledgment. The cross-sectional design precludes causal inference regarding whether observed neuromuscular alterations precede or result from CLBP development. A longitudinal investigation is necessary to establish temporal relationships among loading exposure, pain onset, and motor adaptation. The sample comprised exclusively young adult males (ages 23–28 years) with moderate disability (ODI 20–40%), restricting generalizability to female populations, older adults, severe disability phenotypes, and non-specific CLBP subtypes with different motor control characteristics [47]. This homogeneous sample was selected to minimize confounding from age-related neuromuscular decline and sex-related morphological differences [55], thereby enabling detection of CLBP-specific adaptations; however, it limits external validity. The absence of three-dimensional kinematic data precludes direct quantification of compensatory postural adjustments (lateral trunk lean, pelvic obliquity) that mediate the documented neuromuscular patterns. The fixed walking cadence (100 beats per minute), while controlling temporal variability, may have suppressed natural compensatory strategies involving gait speed modulation. Load magnitudes (10% and 20% body weight) represent modest occupational demands; dose–response relationships at higher magnitudes require investigation.

Methodological constraints specific to electromyographic assessment warrant consideration. Muscle activation–length relationships reported in Section 3.3 were inferred from gait phase timing rather than measured directly via kinematic assessment of muscle-tendon unit length, which introduces potential error if CLBP participants exhibited altered joint kinematics during stance. This inferential approach provides directional evidence of altered length-dependent recruitment but cannot quantify precise operating lengths. Normalization of EMG amplitudes to maximum voluntary isometric contractions accounts for inter-individual variability in electrode-skin impedance and muscle size [21]. Still, it may introduce systematic bias in CLBP populations if pain-related inhibition suppressed maximal effort during MVIC testing [34]. If MVIC amplitudes were artificially reduced in the CLBP group, subsequent normalization would increase walking-trial %MVIC values, potentially exaggerating between-group differences. However, the MVIC protocol employed muscle-specific positioning to minimize lumbar loading and used strong verbal encouragement with visual feedback, procedures that have demonstrated reliability in CLBP populations (test–retest ICC > 0.85) [27]. Furthermore, the observed between-group activation differences (23.3% relative increase) substantially exceed measurement error attributable to normalization variability (typically <10%), suggesting genuine neuromuscular adaptation rather than methodological artifact. Bilateral electrode placement across seven trunk muscles enabled a comprehensive assessment of regional activation patterns, providing multidimensional characterization of motor control strategies despite the absence of three-dimensional kinematic data [30,35].

The study’s strengths include the use of validated, standardized electromyographic protocols with rigorous impedance control and normalization procedures, enhancing measurement precision and between-subject comparability [31]. The within-subject repeated-measures design optimized statistical power while controlling for inter-individual variability. The inclusion of multiple trunk muscles across bilateral anatomical quadrants provided a comprehensive characterization of regional activation patterns. The combination of kinetic and electromyographic measurements enabled integrated assessment of external mechanical demands and internal neuromuscular responses.

Future research should investigate longitudinal trajectories of neuromuscular adaptation during prolonged exposure to asymmetric load carriage, identifying critical thresholds for fatigue-related performance decrements and pain exacerbation. Intervention trials evaluating the efficacy of load distribution optimization, strengthening protocols targeting identified deficits, and motor control retraining in reducing pain and improving functional capacity would advance translation of these findings into clinical practice. Investigation of individual-difference factors (e.g., pain phenotype, psychological factors, movement variability) that moderate neuromuscular responses to asymmetric loading would inform precision medicine approaches to the management of CLBP.

### Practical Recommendations

The present findings support several evidence-based recommendations for occupational health practitioners and rehabilitation specialists managing individuals with CLBP during load carriage. Asymmetric loading should be minimized whenever feasible, particularly at loads exceeding 15% body weight, where disproportionate mechanical and neuromuscular demands emerge. When asymmetric carriage is unavoidable, load redistribution strategies (e.g., frequent side-switching, bilateral partitioning of total load) should be implemented to reduce cumulative unilateral exposure [11]. Rehabilitation programs should incorporate targeted strengthening and endurance training for the lumbar multifidus and erector spinae, given the elevated activation demands documented under asymmetric conditions [52]. Neuromuscular re-education interventions employing real-time biofeedback may address excessive activation of trunk muscles and facilitate more efficient motor strategies [51]. Proprioceptive training emphasizing recalibration of length-tension relationships through controlled lengthening exercises and position-matching tasks may remediate the altered activation–length profiles characteristic of CLBP populations [49]. Occupational task analysis should quantify asymmetric loading exposure and incorporate these metrics into ergonomic risk assessment frameworks alongside traditional load-magnitude and frequency parameters.

## 5. Conclusions

This investigation demonstrates associations between asymmetric load carriage and elevated mechanical and neuromuscular demands during walking, with amplified responses observed in individuals with CLBP. The documented alterations in trunk muscle activation patterns, including global hyperactivation, phase-dependent recruitment abnormalities, and disrupted activation–length relationships, provide quantitative evidence of fundamental neuromuscular dysfunction in this population. The nonlinear interaction between load magnitude and distribution asymmetry highlights the importance of considering both parameters in occupational exposure assessment and ergonomic intervention design. These findings advance mechanistic understanding of load-carriage biomechanics in CLBP and provide an empirical foundation for evidence-based interventions targeting load distribution optimization, targeted muscle strengthening, and motor control restoration to mitigate pain and functional disability in occupational and rehabilitation contexts.

## Figures and Tables

**Table 1:** Normalized electromyographic activation (%MVIC) of left-side trunk muscles under variable loading conditions in CLBP and control participants.

Muscle	CLBP Group					Control Group					Statistical Effects (*p*-Value)			
	No Load	As 10%	S 10%	As 20%	S 20%	No Load	As 10%	S 10%	As 20%	S 20%	G	P	LC	G×P×LC
**LEO**	0.126 ^d^	0.187 ^b^	0.154 ^c^	0.243 ^a^	0.216 ^a^	0.100 ^d^	0.172 ^b^	0.144 ^c^	0.214 ^a^	0.190 ^b^	0.001	0.001	0.001	0.059
**LIO**	0.125 ^c^	0.188 ^b^	0.161 ^b^	0.237 ^a^	0.222 ^a^	0.094	0.173 ^b^	0.134 ^c^	0.220 ^a^	0.187 ^a^	0.001	0.001	0.001	0.052
**LLD**	0.100 ^c^	0.174 ^b^	0.134 ^c^	0.215 ^a^	0.188 ^a^	0.075	0.152 ^bc^	0.117 ^c^	0.195 ^a^	0.175 ^b^	0.001	0.001	0.001	0.136
**LLE**	0.154 ^c^	0.230 ^a^	0.201 ^b^	0.282 ^a^	0.256 ^a^	0.140 ^c^	0.220 ^ab^	0.183 ^b^	0.262 ^a^	0.230 ^a^	0.001	0.001	0.001	0.451
**LM**	0.143 ^c^	0.219 ^ab^	0.177 ^b^	0.263 ^a^	0.225 ^a^	0.121 ^c^	0.192 ^b^	0.158 ^bc^	0.239 ^a^	0.211 ^ab^	0.001	0.001	0.001	0.595
**LRA**	0.120 ^c^	0.193 ^b^	0.161 ^b^	0.240 ^a^	0.211 ^a^	0.099	0.171 ^b^	0.136 ^c^	0.222 ^a^	0.187 ^b^	0.001	0.001	0.001	0.883
**LTF**	0.125 ^c^	0.186 ^b^	0.156 ^bc^	0.244 ^a^	0.206 ^a^	0.095 ^c^	0.169 ^b^	0.137 ^c^	0.210 ^a^	0.185 ^b^	0.001	0.001	0.001	0.114

**LEO**: Left External Oblique; **LIO**: Left Internal Oblique; **LLD**: Left Latissimus Dorsi; **LLE**: Left Lum-bar Erector Spinae; **LM**: Left Multifidus; **LRA**: Left Rectus Abdominis; **LTF**: Left Thoracolumbar Fascia; **G**: experiment groups (injured and control); **P**: Weight lifting position (none, asymmetric and symmetric); **LC**: load condition (none, 10% of body weight and 20% of body weight); **As**: Asymmetric; **S**: Symmetric.. ^a^ Identifies the loading condition(s) producing the highest mean %MVIC value within a given row (specific muscle × group combination). When multiple conditions share a superscript “a”, they do not differ significantly from one another (*p* ≥ 0.05), but all produce significantly higher activation than conditions marked with other letters (b, c, d; all pairwise *p* < 0.05). ^b^ Identifies the loading condition(s) producing intermediate-high mean %MVIC values. Conditions marked “b” do not differ significantly from one another (*p* ≥ 0.05) but produce significantly lower activation than “a” conditions (*p* < 0.05) and significantly higher activation than “c” or “d” conditions (*p* < 0.05). ^c^ Identifies the loading condition(s) producing intermediate mean %MVIC values. Conditions marked “c” do not differ significantly from one another (*p* ≥ 0.05) but produce significantly lower activation than “a” and “b” conditions (*p* < 0.05) and significantly higher activation than “d” conditions (*p* < 0.05). ^d^ Identifies the loading condition(s) producing the lowest mean %MVIC value within a given row. Conditions marked “d” do not differ significantly from one another (*p* ≥ 0.05) but produce significantly lower activation than all other lettered conditions (a, b, c; all pairwise *p* < 0.05).

**Table 2:** Normalized electromyographic activation (%MVIC) of right-side trunk muscles under variable loading conditions in CLBP and control participants.

Muscle	CLBP Group					Control Group					Statistical Effects (*p*-Value)			
	No Load	As 10%	S 10%	As 20%	S 20%	No Load	As 10%	S 10%	As 20%	S 20%	G	P	LC	G×P×LC
**REO**	0.129 ^e^	0.197 ^bc^	0.158 ^d^	0.236 ^a^	0.214 ^b^	0.104 ^e^	0.170 ^c^	0.132 ^d^	0.212 ^b^	0.189 ^bc^	0.001	0.001	0.001	0.156
**RIO**	0.122 ^e^	0.186 ^bc^	0.163 ^d^	0.231 ^a^	0.210 ^b^	0.097 ^e^	0.173 ^c^	0.140 ^d^	0.213 ^b^	0.196 ^bc^	0.001	0.001	0.001	0.351
**RLD**	0.097 ^e^	0.169 ^bc^	0.142 ^d^	0.220 ^a^	0.193 ^b^	0.079 ^e^	0.145 ^c^	0.117 ^d^	0.190 ^b^	0.169 ^bc^	0.001	0.001	0.001	0.647
**RLE**	0.167 ^e^	0.227 ^b^	0.199 ^cd^	0.276 ^a^	0.253 ^a^	0.142 ^d^	0.210 ^b^	0.184 ^c^	0.258 ^b^	0.236 ^b^	0.001	0.001	0.001	0.460
**RM**	0.139 ^de^	0.215 ^bc^	0.181 ^c^	0.260 ^a^	0.231 ^b^	0.125 ^e^	0.196 ^c^	0.158 ^d^	0.243 ^b^	0.212 ^bc^	0.001	0.001	0.001	0.835
**RRA**	0.119 ^e^	0.191 ^bc^	0.165 ^d^	0.239 ^a^	0.212 ^b^	0.097 ^e^	0.172 ^c^	0.144 ^d^	0.215 ^b^	0.197 ^bc^	0.001	0.001	0.001	0.434
**RTF**	0.122 ^e^	0.187 ^bc^	0.122 ^e^	0.239 ^a^	0.159 ^c^	0.105 ^e^	0.175 ^c^	0.146 ^d^	0.213 ^b^	0.190 ^bc^	0.001	0.001	0.001	0.463

**REO**: Right External Oblique; **RIO**: Right Internal Oblique; **RLD**: Right Latissimus Dorsi; **RLE**: Right Lumbar Erector Spinae; **RM**: Right Multifidus; **RRA**: Right Rectus Abdominis; **RTF**: Right Thoracolumbar Fascia; **G**: experiment groups (injured and control); **P**: Weight lifting position (none, asymmetric and symmetric); **LC**: load condition (none, 10 % of body weight and 20 % of body weight); **As**: Asymmetric; **S**: Symmetric.. ^a^ Identifies the loading condition(s) producing the highest mean %MVIC value within a given row (specific muscle × group combination). When multiple conditions share a superscript “a”, they do not differ significantly from one another (*p* ≥ 0.05), but all produce significantly higher activation than conditions marked with other letters (b, c, d, e; all pairwise *p* < 0.05). ^b^ Identifies the loading condition(s) producing intermediate-high mean %MVIC values. Conditions marked “b” do not differ significantly from one another (*p* ≥ 0.05) but produce significantly lower activation than “a” conditions (*p* < 0.05) and significantly higher activation than “c”, “d”, or “e” conditions (*p* < 0.05). ^c^ Identifies the loading condition(s) producing intermediate mean %MVIC values. Conditions marked “c” do not differ significantly from one another (*p* ≥ 0.05) but produce significantly lower activation than “a” and “b” conditions (*p* < 0.05) and significantly higher activation than “d” or “e” conditions (*p* < 0.05). ^d^ Identifies the loading condition(s) producing intermediate-low mean %MVIC values. Conditions marked “d” do not differ significantly from one another (*p* ≥ 0.05) but produce significantly lower activation than “a”, “b”, and “c” conditions (*p* < 0.05) and significantly higher activation than “e” conditions (*p* < 0.05). ^e^ Identifies the loading condition(s) producing the lowest mean %MVIC value within a given row. Conditions marked “e” do not differ significantly from one another (*p* ≥ 0.05) but produce significantly lower activation than all other lettered conditions (a, b, c, d; all pairwise *p* < 0.05).

## Data Availability

The raw data supporting the conclusions of this article will be made available upon request by the corresponding authors.
